# Social Noise Exposure in a Sample of Slovak University Students

**DOI:** 10.3390/ijerph17010324

**Published:** 2020-01-02

**Authors:** Alexandra Filova, Jana Jurkovicova, Katarina Hirosova, Diana Vondrova, Barbora Filova, Martin Samohyl, Jana Babjakova, Juraj Stofko, Lubica Argalasova

**Affiliations:** 1 Institute of Hygiene, Faculty of Medicine, Comenius University, 81499 Bratislava, Slovakia; alexandra.filova@fmed.uniba.sk (A.F.); jana.jurkovicova@fmed.uniba.sk (J.J.); katarina.hirosova@fmed.uniba.sk (K.H.); diana.vondrova@fmed.uniba.sk (D.V.); martin.samohyl@fmed.uniba.sk (M.S.); jana.babjakova@fmed.uniba.sk (J.B.); 2 Institute of Medical Physics, Biophysics, Informatics and Telemedicine, Faculty of Medicine, Comenius University, Sasinkova 2, 81372 Bratislava, Slovakia; barbora.filova@fmed.uniba.sk; 3 Institute of Physiotherapy, Balneology and Medical Rehabilitation, University of Ss. Cyril and Methodius in Trnava, 91701 Trnava, Slovakia; juraj.stofko@gmail.com

**Keywords:** social noise, auditory, non-auditory noise effects, personal music players, university students

## Abstract

Purpose: Social noise exposure is currently an emerging problem in adolescents and young adults. Various leisure time activities may be responsible for hearing impairment (temporary or permanent hearing threshold shift or hearing loss). The study aimed to quantify environmental noise from various sources—voluntary (social) noise (personal music players (PMPs), high-intensity noise exposure events), and road traffic noise and to detect hearing disorders in relation to individual listening to PMPs in the sample of young adults living and studying in Bratislava, the capital city of Slovakia. Methods: The study included 1003 university students (306 men and 697 women, average age 23.1 ± 2) living in Bratislava for 4 or more years; 347 lived in the student housing facility exposed to road traffic noise (L*_Aeq_* = 67.6 dB) and 656 in the control one (L*_Aeq_* = 53.4 dB). Respondents completed a validated ICBEN 5-grade scale “noise annoyance questionnaire”. In the exposed group a significant source of annoyance was road traffic noise (*p* < 0.001), noise from entertainment facilities (*p* < 0.001), industrial noise (*p* < 0.001), and noise from neighboring flats (*p* = 0.003). The exposure to PMPs was objectified by the conversion of the subjective evaluation of the volume setting and duration. With the cooperation of the Ear, Nose and Throat (ENT)specialist, we arranged audiometric examinations on the pilot sample of 41 volunteers. Results: From the total sample of respondents, 79.2% reported the use of a PMP in the course of the last week, and the average time was 285 min. There was a significant difference in PMP use between the road traffic noise-exposed (85.6%) and the control group (75.8%) (*p* = 0.01). Among PMP users 30.7% exceeded the lower action value (LAV) for industry (L*_Aeq_*_,8*h*_ = 80 dB). On a pilot sample of volunteers (*n* = 41), audiometry testing was performed indicating a hearing threshold shift at higher frequencies in 22% of subjects. Conclusions: The results of the study on a sample of young healthy individuals showed the importance of exposure to social noise as well as to road traffic noise and the need for prevention and intervention.

## 1. Introduction

Noise has attracted widespread attention as a significant environmental and occupational health concern. The environmental noise has often been referred to as the “forgotten pollutant” but is now recognized as an environmental and public health issue that needs to be addressed in modern society. It concerns mainly the population living in urban areas but also in rural areas with the main sources being road, rail and air traffic, industries, construction and public work, and the neighborhood [1,2,3,4]. According to the World Health Organization (WHO), excessive environmental noise interferes with people’s daily activities at school, at work, at home, and during leisure time. It can disturb sleep, cause cardiovascular and psychophysiological effects, reduce performance, and provoke annoyance responses and changes in social behavior [1,2,3,4,5,6,7,8,9,10,11,12]. According to the environmental burden of disease (EBD) approach, traffic noise exposure features cause an annual loss of 31 disability-adjusted life years per 100,000 population in the WHO European region [1]. 

In addition to exposure to environmental noise, voluntary or social noise exposure is very important nowadays. This type of exposure is currently a major problem, especially among adolescents and young adults. Various leisure time activities (often listening to music on high-volume level with personal music players (PMPs) and regular visiting of events with high noise exposure intensity) can be responsible for auditory (temporary or permanent hearing threshold shift, hearing loss) or non-auditory effects on individuals (annoyance, sleeping disorders, nervousness, irritability, high blood pressure) [1,3,7,13].

Hearing loss due to noise exposure is the most common cause of deafness and hearing impairment. Although genetics and advanced age are the main risk factors, temporary and permanent hearing impairment is increasingly common among young adults and children, mainly due to increased exposure from personal music players and noisy leisure time activities [7].

Excessive listening to personal music players often leads to the shift of hearing thresholds. The development of new technologies brings to market devices that can produce sound at high levels and thus cause considerable damage to the hearing organ. Hearing damage is the most common in people who listen to music on PMPs for more than 5 years. In Europe, there are 2.5 to 10 million listeners of PMPs, most often children and young people. Estimated unit sales ranged between 184–246 million for all portable audio devices and between 124–165 million for MP3 players [14]. 

Listening to loud music from portable music players at least five hours a week exceeds the noise standards to which workers may be exposed in the noisiest industrial enterprises. By inserting the earphones into the ear, the hearing canal closes, and the intensity of the sound/music increases. These characteristics become increasingly detrimental with longer and louder PMP listening. This relates not only to music but also to any sound with a high volume [15,16].

The study aimed to quantify environmental noise from various sources—voluntary (social) noise (personal music players (PMPs), high-intensity noise exposure events) and road traffic noise and to detect hearing disorders in relation to individuals listening to PMPs in the sample of young healthy Slovak individuals—university students living and studying in Bratislava, the capital city of Slovakia.

## 2. Materials and Methods

The research was conducted at the Institute of Hygiene Faculty of Medicine Comenius University in Bratislava, Slovakia. We used the method of subjective evaluation using a standardized anonymous questionnaire and methods of objectification by direct measurement of noise levels using the hand-held sound analyzer with the frequency analysis software. For screening of hearing, we used threshold tonal audiometry, which determines the hearing threshold for pure tones for both left and right ears.

### 2.1. Study Subjects

The sample comprised 1003 subjects, 697 (69%) females and 306 (31%) males. The average age was 23.13 ± 2 years. The source population (*n* = 1500) was composed of students enrolled at the Faculty of Medicine, Comenius University. The respondents represented a homogenous sample of young healthy individuals of comparable age, education, and lifestyle (Table 1). The response rate was 90%. Only those students living in Bratislava, the Slovakian capital, for at least 4 years (permanent or temporary residence) were eligible to participate in the study (*n* = 1100). Subjects from the exposed group stayed at the college dormitory Druzba at Comenius University (CU). This group included 347 (34.6%) subjects. Subjects from the control group stayed at the college dormitory of Ludovit Stur, Stare Grunty, CU. This group included 656 (65%) university students. The exposed housing facility and student dormitory is situated near the major transportation route, the main thoroughfare with railway transport; the control housing facility and student dormitory in a quiet area with surrounding greenery. Subjects significantly differed by age, by traffic noise exposure, flat location in relation to noise exposure, position of flat in floor height, length of stay in the given area, orientation and the type of windows, satisfaction with flat surrounding, and the use of personal music players (PMPs) (Table 1).

### 2.2. Noise Annoyance Questionnaire

We used a validated methodology for subjective evaluation of noise annoyance and interference with various activities. We assessed the quality of sleep and psychosocial well-being in relation to noise exposure. Respondents filled in validated “noise annoyance questionnaires”, using a standard 5-point scale (0 = not at all, 1 = slightly, 2 = moderately, 3 = very, 4 = extremely) developed and recommended by experts from the The International Commission on the Biological Effects of Noise (ICBEN) team [17,18]. We focused mainly on sleep disturbance, noise annoyance from various sources, and interference with various activities. Questionnaires and objective examinations were voluntary for each of the respondents. Study participants gave their informed consent to use the information for research purposes. 

### 2.3. Exposure Assessment

We used the method of objectification by direct measurement of noise levels using a hand-held sound analyzer Brüel–Kjaer 2250 with the frequency analysis software. We measured equivalent sound levels in the exposed and also in the control area. All measurements were recorded according to the valid Slovak legislation during the time intervals from 17:00–18:00 and from 20:00–21:00 in the exposed and at the same time in the control area. [18] This time interval was chosen to record the afternoon traffic peak and to detect the time most annoying for students and for their activities (studying, watching TV, talking, and falling asleep). Measurements were recorded during the spring period at working days (Tuesday) two times on each site.

### 2.4. Exposure from Social Noise

In the estimation of exposure from PMPs, we used the methodology of Portnuff et al. [19,20]. Respondents rated subjective intensity and frequency of exposure to personal music players (PMPs). We examined what type of headphones they prefer (earphones or headphones) and at what volume level they listen to their PMPs. They also rated how often they attend events with high-intensity noise exposure (e.g., rock concerts, discos, sports events) and how often they do noisy housework and whether they play a musical instrument). 

### 2.5. Hearing Examination

The hearing examination was provided for volunteers in the cooperation with Ear, Nose and Throat (ENT) specialists in the out-patient Department for Otorhinolaryngology in Bratislava. The students were examined by a subjective audiometric method in which the hearing was checked by the electro-acoustic device-audiometry. The basic examination of the threshold tone audiometry established a threshold hearing for pure tones (lowest stimulus intensity). For determining the hearing threshold for pure tones, the diagnostic audiometer MAICO MA 52 was used. This audiometer allows investigating overhead lines at frequencies of 250–6000 Hz and bone conduction at frequencies 500, 1000, 2000, and 4000 Hz. During the examination, the subjects were set into a quiet chamber, the audiometric cabin in the medical office. The chamber is used for audiometric testing tone audiometry, in particular for determining the hearing threshold for pure tones and meeting the essential parameters, especially, the desired attenuation of the intensity of sounds. It corresponds to ISO 266: 1997 and ISO 389–3: 2016.

### 2.6. Statistical Analysis

To evaluate the results, we used the methods of descriptive and analytical statistics to identify mutual associations between lifestyle factors, psychosocial, biological, behavioral and environmental factors using statistical package Epi InfoTM software, version 7.1.5.0, Atlanta, GA, USA, and SPSS, version 24 (International Business Machines Corp.; New Orchard Road; Armonk, NY, USA).

## 3. Results

The monitoring of sound levels in the exposed area showed levels above the national and international limits in the afternoon and in the evening time interval 17:00–18:00 and 20:00–21:00 (L*_Aeq_* = 67.6; 64.7 dB). Sound levels in the control area were significantly lower (*p* < 0.001) (L*_Aeq_* = 53.4; 54.3 dB) [9,18]. The higher sound levels in the evening interval in the control area could be due to the other noise sources (e.g., entertainment facilities) (Table 2).

In addition to monitoring the noise levels, we evaluated the traffic flow (Table 3 and Table 4). In the exposed area, passenger cars dominated, followed by trams, buses, and motorcycles. The transport intensity corresponds to the results of noise level measurements. In the control area, most of the vehicles were passenger cars, then buses, trucks, and motorcycles.

The indicators estimated from Bratislava strategic noise map were L_DE*N*_ = 66 ± 2 dB vs. L_DE*N*_ = 56 ± 4 dB (*p* < 0.05) (http://www.laermkarten.de/bratislava/) [21].

**Figure 1:**
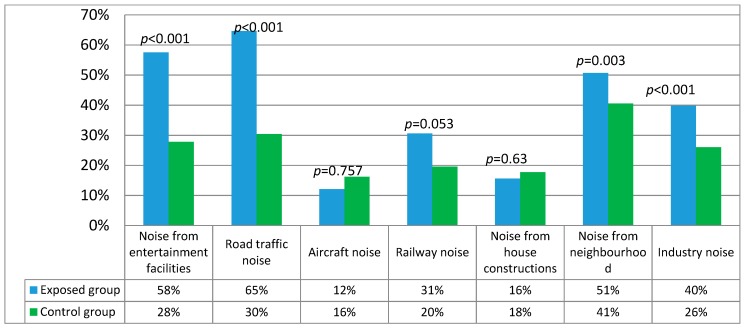
Comparison of noise annoyance from different sources between the exposed group to road traffic noise and the control group.

When comparing the results of noise annoyance from different sources between the exposed group to road traffic noise and the control group, a significant source of annoyance was road traffic noise (*p* < 0.001), noise from entertainment facilities (*p* < 0.001), industrial noise (*p* < 0.001), and noise from neighboring flats (*p* = 0.003) (Figure 1). Based on noise annoyance risks, a significant risk factor in the exposed group was road traffic noise (OR_MH_ = 3.89; 95% confidence interval, CI, = 3.14–4.81), noise from entertainment facilities (OR_MH_ = 3.58; 95% CI = 2.92–4.38), industrial noise (OR_MH_ = 1.54; 95% CI = 1.33–1.8), neighboring flats noise (OR_MH_ = 1.6; 95% CI = 1.32–1.93) and railway noise (OR = 1.52; 95% CI = 1.23–1.87). There was no significant difference concerning noise annoyance risks from house constructions and aircraft noise.

From the total sample of respondents, 794 (79%) students reported listening to PMPs in the last week for an average time of 285 min. There was a significant difference in PMP use between the exposed group to road traffic noise (86%) and the control group (76%) (*p* = 0.0002) and also in the duration of listening to PMPs in minutes (286 ± 367 vs. 292 ± 367) (*p* < 0.55), but it was not significant between genders (*p* = 0.08).

About 16% of students listen to the music on the loudness level 4 (they cannot hear the speech or even the traffic), and 86.5% use earbuds. There was no significant difference between the loudness level of PMP or in the duration of time spent at most events with high noise exposure between the exposed and control group.

The significant difference was in the type of headphones; earbuds are more often used by students from the exposed area (more than 90% of students) (*p* = 0.01). Earbud-inserted phone types are more harmful according to SCENIHR (2008) and increase the sound level by 7–9 dB [14]. Based on the subjective assessment, the reduced hearing ability indicated 25.7% of subjects from the exposed location and 22% of subjects in the control location (*p* = 0.23). The presence of subjective hearing impairment of PMP users was not significantly higher (24%), compared to the non-PMP users (20.7%) (*p* = 0.4).

**Figure 2:**
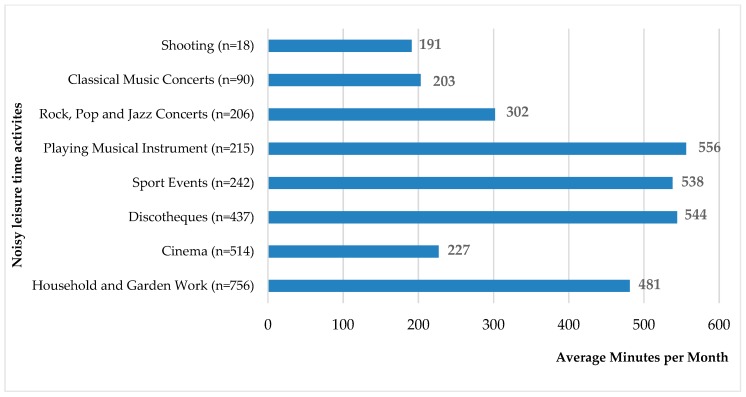
The number of students taking part in leisure time activities (average minutes per month).

From activities with high intensity of noise exposure, most students took part in household and garden work (*n* = 756), where they spent on average 481 min. The second most frequent activity was visiting a cinema (*n* = 514), where students spent on average 227 min per month. The third one was visiting discotheques (*n* = 437), where they spent on average 544 min per month. The fourth most preferred activity was visiting sports events (*n* = 242) where they spent on average 538 min per month. Students spent 556 min playing a musical instrument on average (*n* = 215). Many subjects (*n* = 206) attended rock, pop, or jazz concerts, where they spent on average 302 min per month. Students spent 203 min visiting classical music concerts (*n* = 90) and 191 min in sport shooting (*n* = 18) (Figure 2).

Students from the exposed group to road traffic noise spent in the cinema about 244 min per month, compared with subjects in the control group who spent in the cinema on average 170 min per month (*p* = 0.08). There was no significant difference between the duration of time spent at the other events with high noise exposure between the exposed and the control group.

In cooperation with the ENT specialist, we performed audiometric testing on a pilot sample of volunteers, university students (*n* = 41), in which we found indicated hearing threshold shift in higher frequencies in 22% of subjects.

The prevalence of audiometric hearing impairment is defined as a threshold average greater than 20 dB hearing level in adults [22]; in children it is 16 dB according to Niskar et al. [23].

**Figure 3:**
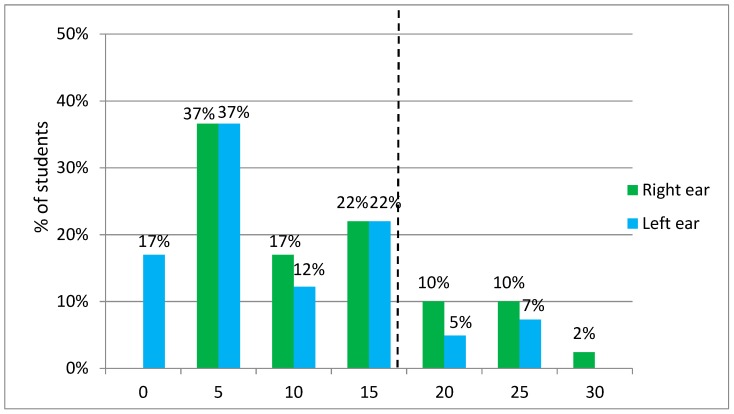
Hearing thresholds for pure tones at 8000 Hz—right and left ear (*n* = 41). Note: On the right side of the dotted line are subjects exceeding the threshold (16 dB).

On frequency 8000 Hz this threshold exceeded nine (22%) subjects on the right ear and five (12%) subjects on the left ear (Figure 3).

We also calculated hearing threshold averages: Low-frequency average (500, 1000, and 2000 Hz) for the left and right ear, high-frequency average on the (3000, 4000, 6000, and 8000 Hz) for the left and right ear. Normal hearing was defined as 0–15 dB, slight loss 16–25 dB, mild loss 26–40 dB, moderate loss 41–65 dB, and severe loss 66–95 dB. Due to the small group of young healthy volunteers, the results of audiometric tests were only in the first three categories (Table 5 and Table 6).

The examined subjects had impaired hearing in the higher frequencies of 6000 Hz and 8000 Hz bilaterally and also in the lower frequencies of 1000 Hz in the right ear and 2000 Hz bilaterally (Table 5 and Table 6).

**Figure 4:**
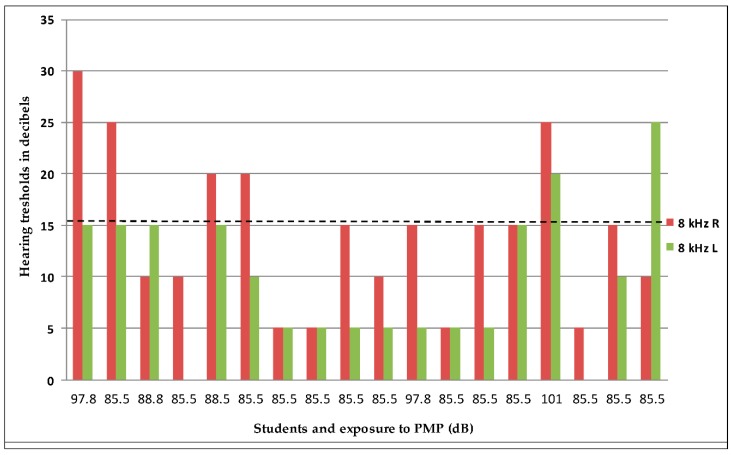
Students listening to PMPs on high sound levels and their hearing thresholds at 8 kHz. Note: Above the dotted line are subjects exceeding the threshold (16 dB), R—right ear, L—left ear.

Of those volunteers who attended audiometric testing, more than 43% exceeded the lower action value (LAV) for industry (80 dB) by listening to their PMPs. In this group, the decreased hearing in 28% of subjects was found (Figure 4).

## 4. Discussion

In our study on 1003 university students from Bratislava, up to 794 students (79.2%) reported listening to personal music players (PMPs) in the last week, with an average listening time of 285 min, and 26% of PMP users exceeded LAV (lower action values of noise at work = 80 dB). In the exposed group, 85.6% of respondents were listening to PMPs compared to the control group, where PMPs were used by 75.8% of students. This difference was significant on *p* < 0.001. We can conclude that, apart from traffic noise, the students from the exposed area were also exposed to social noise from personal music players. However, the length and intensity of PMP listening did not differ significantly between these two groups.

On the basis of audiometric tests in a pilot sample of 41 volunteers, we found a threshold shift at high frequencies in 22% of examined subjects. 

This vulnerable group of young people from the age of 20–30 years is exposed to many noise sources (from traffic to construction facilities and neighbors, to social noise events with high intensity of noise exposure in addition to frequent listening to PMPs on high volume setting). This group requires special attention in the prevention of hearing impairment. Leisure time activities with high-intensity noise exposure are comparable to occupational noise [24]. Various leisure-time activities can be responsible for hearing disorders (temporary or permanent shift of auditory threshold, hearing loss). Exposure to this noise source is compared to the lower noise action value at work. The limit under the Directive 2003/10/EC-noise level A—80 dB for 40 h—is reached after less than 30 min per week [25]. There are also personal music players (PMPs), which at high volume (above 89 dB) reach the noise exposure equivalent to the lower action value after 5 h per week. We can, therefore, conclude that personal music players represent a risk to hearing at high sound pressure levels during long-term exposure. Around 2.5 to 10 million citizens use PMPs so often and so loudly that they risk hearing loss after five years of use [14,26].

In the most recent studies, the authors recommended the use of high-frequency audiometry and evoked otoacoustic emissions (EOAE) (namely transient-evoked otoacoustic emissions (TEOAEs) and distortion-product OAEs (DPOAEs)) [15]. OAEs (otoacoustic emissions) are the sounds produced by the inner ear and recorded by a miniature microphone in the external auditory canal. In the diagnostics, only evoked OAEs can be used. TEOAEs (transient OAEs) are emissions (audible responses) from the cochlea recorded by a miniature microphone in the probe in an external auditory canal and are caused by small sound impulses (clicks) broadcasted through the probe in an external ear (probe with two channels). DPOAEs are generated when the cochlea is stimulated simultaneously by two tones with different frequencies. This frequency specificity allows the monitoring of changes in the cochlea and early detection of hearing impairment (noise, ototoxic drugs, Ménière’s disease). EOAEs reflect the integrity of cochlear hair cells and are useful in the pre-clinical detection of hearing impairment. This screening tool is therefore important in younger individuals [22,27].

Our results are consistent with the results of other studies, reporting 88–90% of adolescents and young adults listening to PMPs through headphones, especially earplugs. Recent publications evaluated increased risk of hearing disorders in relation to the listening to PMPs and the current incidence of hearing loss and tinnitus. The prevalence of hearing impairment caused by noise in adolescents and young adults was 17% and 29% in Europe [7]. Hearing threshold shift ≥25 dB at frequencies of one or more occurred in 7.3% of 177 subjects in Malaysia [28]. In a large sample of students from ninth-grade primary schools in Bavaria (*n* = 1843), the prevalence of audiometric notches was only 2.4% and indicated the need to follow this sample longitudinally or focus on the older age groups, such as university students [29]. In the study on 34 medical students, regular PMP listeners, the authors observed the immediate effect of short-term PMP listening on DPOAE at 9–12 kHz [30].

Students in the group exposed to road traffic noise were listening to PMPs more often than students in the control group (possibly trying to mask the effect of road traffic noise or environmental noise from the other sources). However, the volume of listening to PMPs was not significantly different between those two groups, and neither was the duration of time spent at leisure time events or other activities with high noise exposure. These results are difficult to compare with the other relevant literature because we are not aware of any studies that have recently researched such associations.

However, the study has several limitations. The design is cross-sectional, taking place at a single point in time and does not allow the discovery of a causal relationship between cause and effect. Longitudinal design controls the time factor and the influence on the development of the analyzed facts, so undertaking this type of study in the future should be considered. Recall bias resulting from overestimating or underestimating the level of subjective noise annoyance reported by respondents may occur. Interviewer bias might occur because questionnaires were administered by persons involved in the study and familiar with the topic. Aircraft noise is not an issue in this study because the study population lived far from the airport, located on the other side of the city. The pilot sample of 41 volunteers undergoing audiometric examination is too low, and we would like to enlarge the sample to 100 subjects in the future.

Hearing loss due to noise is the most common cause of deafness and hearing impairment. Although treatment options are limited, for most people suffering from noise-induced hearing loss, several modifiable health habits should start to grow from childhood and can prevent or delay the onset of hearing impairment.

The studies on hearing loss of youth and the identification of causes of hearing loss in adolescents are very important in order to develop additional precautions. It is also important to determine which groups of those young and healthy individuals are particularly vulnerable to effectively target preventive measures.

## 5. Conclusions

The results of the study on a sample of young healthy individuals showed the importance of exposure to social noise as well as to road traffic noise and the need for prevention and intervention.

Personal music players are now available to everyone in the form of MP3 players or smartphones, and listening to music through PMPs is extremely popular, especially among young adults. Leisure time activities with high-intensity noise exposure are also very popular (rock concerts, discotheques, cinema) as well as sports activities and household or garden work. All these activities may be responsible for an early hearing impairment (temporary or permanent hearing threshold shift, hearing loss) starting at a younger age. 

After the finalization of the study results, we would like to formulate the proposals and interventional procedures and effectively target the preventive measures (education, the use of noise-canceling headphones for PMP users) in the vulnerable groups of teenagers and young adults and their parents and teachers as well.

## Figures and Tables

**Table 1:** Characteristics of the students’ sample.

Variable	Exposed Group * (*n* = 347)		Control Group * (*n* = 656)		*p*-Value
	*n*	%	*n*	%	
**Gender**					0.28
Male	102	29	204	31	
Female	245	71	452	69	
Age **					
Male	23 ± 2 22.63 ± 0.95 years		23 ± 2		0.76
Female	23 ± 2		23 ± 2		
**The use of a PMP in the last week (subjectively)**					
No	50	14	159	24	0.0002
Yes	297	86	497	76	
**The loudness of PMP music**					
1 Not louder than speech	43	14	96	19	0.0002
2 Could hear talking	129	43	181	36	
3 Could hear traffic	84	28	145	29	
4 Could not hear either talking	44	15	82	16	
or traffic					
**Type of headphones**					
Earbuds	273	91	428	84	0.01
Headset	27	9	82	16	
Other noisy events and activities (min/month) ***					
Playing a music instrument	548 ± 884		551 ± 887		0.38
Visit to the cinema	228 ± 359		228± 359		0.32
Visit to classical concerts	203 ± 238		205 ± 235		0.26
Visit to rock, pop, jazz concerts	305 ± 464		302 ± 458		0.06
Visit to discotheques	544 ± 776		543 ± 767		0.30
Visit to sport events	483 ± 777		483 ± 777		0.89

* There are missing values for each variable category. ** Average age in the sample (arithmetic mean ± standard deviation). *** Average number of minutes per month (arithmetic mean ± standard deviation). PMP, personal music player.

**Table 2:** Sound levels in the exposed and control housing facility.

Time Intervals (h)	Sound Levels in the Exposed Housing Facility (*L_Aeq_*) dB	Sound Levels in the Control Housing Facility (*L_Aeq_*) dB
17:00–18:00	67.6	53.4
20:00–21:00	64.7	54.3

**Table 3:** Traffic flow in the exposed area at different time intervals.

Time Interval	17:00–18:00	20:00–21:00
**Automobiles**	6470	3770
**Trams**	110	60
**Motorcycles**	0	24
**Buses**	40	24
**Lorries**	20	0

**Table 4:** Traffic flow in the control area at different time intervals.

Time Interval	17:00–18:00	20:00–21:00
**Automobiles**	420	204
**Trams**	0	0
**Motorcycles**	0	0
**Buses**	12	12
**Lorries**	12	0

**Table 5:** Prevalence of hearing threshold shifts, right ear.

Frequency, kHz	0–15 dB (Normal)	16–25 dB (Slight)	25 or More dB (Mild)
0.5	100%	0%	0%
1	98%	2%	0%
2	98%	2%	0%
4	100%	0%	0%
6	98%	2%	0%
8	78%	20%	2%

**Table 6:** Prevalence of hearing threshold shifts, left ear.

Frequency, kHz	0–15 dB (Normal)	16–25 dB (Slight)	25 or More dB (Mild)
0.5	100%	0%	0%
1	100%	0%	0%
2	95%	5%	0%
4	100%	0%	0%
6	93%	7%	0%
8	88%	12%	0%
